# Stand Composition, Tree Proximity and Size Have Minimal Effects on Leaf Function of Coexisting Aspen and Subalpine Fir

**DOI:** 10.1371/journal.pone.0154395

**Published:** 2016-04-28

**Authors:** Aaron C. Rhodes, Trevor Barney, Samuel B. St. Clair

**Affiliations:** Plant and Wildlife Sciences, Brigham Young University, Provo, Utah, United States of America; US Geological Survey, UNITED STATES

## Abstract

Forest structural heterogeneity due to species composition, spatial relationships and tree size are widely studied patterns in forest systems, but their impacts on tree function are not as well documented. The objective of this study was to examine how stand composition, tree proximity relationships and tree size influence the leaf functional traits of aspen, an early successional species, and subalpine fir, a climax species. We measured foliar nutrients, nonstructural carbohydrates (aspen only), defense chemistry and xylem water potential of aspen and subalpine fir trees in three size classes growing in close proximity or independently from other trees under three stand conditions: aspen dominant, aspen-conifer mixed, and conifer dominant stands. Close proximity of subalpine fir to aspen reduced aspen’s storage of starch in foliar tissue by 17% suggesting that competition between these species may have small effects on carbon metabolism in aspen leaves. Simple sugar (glucose + sucrose) concentrations in aspen leaves were slightly higher in larger aspen trees than smaller trees. However, no differences were found in stem water potential, foliar concentrations of nitrogen, phosphorus, or secondary defense chemicals of aspen or subalpine fir across the gradients of stand composition, tree proximity or tree size. These results suggest that mechanisms of coexistence allow both aspen and subalpine fir to maintain leaf function across a wide range of stand structural characteristics. For aspen, resource sharing through its clonal root system and high resource storage capacity may partially contribute to its functional stability in mixed aspen-conifer stands.

## Introduction

Stand structural heterogeneity due to species composition, spatial relationships and size structure are widely observed patterns in forest systems that influence plant-plant interactions. Among these interactions, competition and facilitation create feedbacks that alter spatial patterns in plant communities [1, 2, 3]. Plant communities are often influenced by positive plant interactions early in community development in which stress-tolerant colonizer species facilitate seedling establishment and growth of intermediate and late successional species [2, 4]. Once established, plant species can affect environmental conditions in ways that reduce the fitness and establishment of competitor species [5, 6]. Therefore, mid-to-late stage successional species are thought to have an active role in creating stress for competing species [4, 7]. For example, mid-to-late stage successional species can reduce soil resource and light availability, affecting water relations, carbon metabolism, growth and defense chemistry expression in plants [5, 8].

Facilitation and competition are important drivers of plant community assembly. Facilitation often promotes aggregated spatial distributions of plants [1] and increases plant proximity [2, 9], while competition promotes uniform spatial distribution and lower plant densities [10, 11]. There is a vast literature that has examined how forest stand spatial characteristics (i.e. proximity relationships and community composition) influence plant distributions, growth and mortality [3, 12, 13, 14, 15], while fewer studies have examined functional plant responses to these patterns.

Plant size can influence the intensity of competition [11] and defines the plant’s zone of influence [9]. The zone of influence model of plant-plant competition delineates a spatially explicit area in which a plant competes for resources, and these competitive interactions tend to increase as plant size increases [9]. Size dependent zones of influence suggest that smaller plants have less competitive influence on adjacent vegetation, but that relationship can change as plants increase in size over time [3, 12, 16].

Quaking aspen (*Populus tremuloides*, (Michx.)) is an early successional, shade intolerant species and often establishes clonally through root suckering following disturbance [17, 18, 19]. Subalpine fir (*Abies lasiocarpa* (Hook.) Nutt.) is a late successional shade tolerant species that commonly associates with aspen to form mixed seral stands. Subalpine fir aggregates readily at the base of aspen trees through facilitation due to favorable microsites associated with aspen [16, 20]. Close proximity between overstory aspen and subalpine fir (due to this facilitation effect) and high conifer stand abundance increases aspen mortality rates [12]. Lengthened fire-return intervals may intensify antagonistic interactions between aspen and conifer species and could explain the observed shift in stand composition toward increased conifer abundance over time [21]. Antagonistic interactions between competing aspen and subalpine fir may be driven by competition for available resources which can be assessed by measuring leaf resource traits including water potential, nutrient status and carbohydrate status.

Resource availability mediates the selective allocation of resources to growth, defense, and resource storage. [22, 23]. The carbon-nutrient balance theory suggests that resources are first used for primary metabolism (growth and maintenance), and then allocated to energy storage and secondary defense compounds such as phenolics [23]. The theory suggests that plants adapted to high resource environments like early successional stages show physiological plasticity in response to changing resource conditions, while plants adapted to low resource environments may demonstrate greater trait stability under resource limitation [23]. Therefore, competition driven reductions in plant resources may have differential impacts on the leaf functional responses of pioneer and climax species as competition changes with shifts in forest composition and structure through different successional stages. Evaluating resource traits in subalpine fir and aspen foliar tissue may shed light on the physiological processes involved in the spatial relationships between aspen and conifer species.

Here we use mixed aspen-conifer forests as an experimental system to understand how variability in stand composition, tree proximity relationships, and tree size influence the functional responses of aspen and subalpine fir. We examined water relations, foliar nutrition, and defense chemistry of aspen and subalpine fir leaves and nonstructural carbohydrates of aspen leaves. Water relations and foliar nutrients provide information about differences in leaf resource status, while nonstructural carbohydrates are important biomarkers of tree vigor [24]. Relative differences in defense chemical production affects the palatability and susceptibility of plants to herbivory [25]. We hypothesized that greater conifer abundance and proximity to neighboring trees and increasing tree size would create a more competitive environment as evidenced by reductions in stem water potential, foliar nutrients, defense chemistry expression and nonstructural carbohydrates concentrations in foliar tissue with a greater effect on aspen than subalpine fir.

## Materials and Methods

### Study Location

This study was conducted at six field locations spread across the Fishlake National Forest in Central Utah August 1^st^– 3^rd^ of 2012. (38°74′30.38′′N, 111°65′40.53′′W; 38°48′ 21.16′′N, 112°07′59.96′′W; 38°58′85.64′′N, 111°67′ 03.82′′W; 38°76′80.71′′N, 111°68′54.24′′W; 38°69′67.14′′N, 111°53′12.40′′W; 38°53′95.73′′N, 111°68′60.35′′W). Permission to conduct this research on the Fishlake National Forest was granted by Allen Rowley, the Forest Supervisor. These sites ranged from 2700 m to 3000 m in elevation, 6–23 degrees of slope and spanned the geographic range of Fishlake National Forest. At each field site three stands were selected that were immediately adjacent to one another with the following approximate overstory tree compositions: aspen dominant (90% aspen), mixed (50:50, conifer:aspen) and conifer dominant (75% conifer). Stand composition, density, and basal area were quantified using the point-centered quarter method [26] along a 50 meter transect with sample points in 5 meter increments. Average overstory tree composition in the aspen dominant, aspen:conifer mixed and conifer dominant stands across the six field sites were: 90:10, 51:49 and 24:76, respectively. Basal tree area for the aspen, mixed and conifer stands were: 58 ± 12 m^2^ ha^-1^, 76 ± 13 m^2^ ha^-1^, and 59 ± 18 m^2^ ha^-1^. Average stand densities for the aspen, mixed and conifer stands were: 2228 ± 472 stems ha^-1^, 2806 ± 428 stems ha^-1^ and 1978 ± 548 stems ha^-1^. Precipitation data were obtained from the meteorological station at Black Flat-U.M. Ck, 38° 40' 48"N, -111° 35' 60"W [27]. This station is centrally located to the study sites and is at similar elevation. Precipitation in July 2012 was 142 mm, which was well above the 30-year average of 48 mm. However, the preceding months of May and June were particularly dry with 5 mm, and 0 mm of precipitation compared to the 30-year averages of 48 mm and 26 mm, respectively.

### Study Design

In each stand type (aspen dominant, mixed and conifer dominant) at all six field sites, we identified six aspen and six subalpine fir trees. Three mature canopy trees of each species selected for the study were growing in aspen-fir pairs (< 20 cm from each other at the base—which is common because aspen often facilitates subalpine fir establishment and germination [20]) and the remaining three trees of each species were growing independently (>3 m from another tree) with one tree in each of three diameter classes:15–19 cm, 20–24 cm, and 25–30 cm. Sampling and measurements were conducted from 10:00 to 16:00 each day over a three-day period in early August 2012 (1^st^-3^rd^). Sampling order was randomized across treatment classes to avoid any potential diurnal effects. We used a Remington 12 gauge shotgun and pole tree pruner to harvest one branch sample from tree mid-canopies.

### Water Relations and Leaf Collection

Branches were measured immediately after harvesting for xylem water potential. Stem water potential was measured using a pressure bomb (PMS Instrument Company, Albany, OR, USA). Approximately 20 leaves were removed from branches, placed in freezer bags and transported back to the lab between blocks of dry ice. Leaves were stored at -80°C until being freeze dried for 48 hours. Aspen and subalpine fir leaf samples were prepared for chemical analysis by grinding samples in a Wiley Mill (Arthur H. Thomas Scientific, Philadelphia, PA, USA) using a #20 screen.

### Foliar Nutrients–P, N

Total phosphorus was determined by ashing 20 mg of leaf material in a muffle furnace at 495°C for 12 hours. The ash was then dissolved in 2 ml of 100 mM HCl and total phosphorus was determined according to the methods of Murphy and Riley [28]. The results were presented as percentage of dry matter.

Leaf material (100 mg) from aspen and subalpine fir was analyzed for total foliar nitrogen content according to the combustion method [29] using a CN analyzer (Truspec CN Determinator, LECO Cooperation, St. Joseph, Michigan, USA). The results were presented as percentage of dry matter.

### Defense Chemistry–Condensed Tannins, Phenolic Glycosides

Condensed tannins were extracted from 40 mg samples of leaf material by first, suspending the samples in 1 ml of 70% acetone—10 mM ascorbic acid solution. The samples were then vortexed (VorTemp 56, Labnet International Inc., Edison, NJ, USA) at 4°C for 20 minutes, and subsequently centrifuged. Next, the liquid supernatant was transferred to a separate microcentrifuge container and the extraction was repeated a second time. The concentration of condensed tannins was then quantified with a spectrophotometer (SpectraMax Plus 384, MDS, Toronto, Canada), using the modified butanol-HCl method [30], and comparison with purified condensed tannins isolated from aspen leaves as a standard. The results were presented as percentage of dry matter.

Phenolic glycosides were quantified according to the methods of Lindroth 1993 [22]. We extracted two major phenolic glycosides from 40 mg of aspen leaf tissue (salicortin and tremulacin, which constitute 80% of phenolic glycoside mass and are the most active deterrents of herbivory [22]). We placed the leaf tissue in 2 ml screw cap centrifuge tubes and added 1 ml of 80% methanol. We then homogenized the samples in a vortex for 5 minutes and the supernatant was removed and placed in a separate 2 ml centrifuge tube. This process was repeated in order to extract 2 ml of supernatant. We quantified the final concentrations of salicortin and tremulacin from the extracted supernatant using high performance liquid chromatography (Agilent 1100 Series, Santa Clara, CA, USA) with a Luna 2, C18 column (150 x 4.6 mm, 5 μm) at a flow rate of 1 ml/min. We detected compound peaks with a UV lamp at a wavelength of 280 nm. We compared samples to a standard curve from purified salicortin and tremulacin isolated from aspen. Subalpine fir does not produce phenolic glycosides, so subalpine fir samples were not analyzed for phenolic glycosides. The results were presented as percentage of dry matter.

### Nonstructural Carbohydrate Extractions

For each aspen leaf sample, we suspended 20 mg of leaf material in 0.66 ml of 80% ethanol in a 2 ml screw cap microcentrifuge tube and placed them in a heater vortex at 80°C for 20 minutes. We then transferred the supernatant in a separate tube and then repeated the extraction 2 more times. We used the ethanol extract for the glucose and sucrose analysis and the ethanol extracted leaf tissue for starch analysis.

In preparation for the starch analysis, the ethanol extracted tissue was immersed in 1 ml of water in a 2 ml screw cap microcentrifuge tube. The samples were autoclaved for 1 hour at 135°C and then dried overnight in a drying oven at 65°C. The incubation time for enzymatic digestion was insufficient to obtain reliable estimates of nonstructural carbohydrates in subalpine fir foliar tissue, and we had insufficient tissue to reanalyze subalpine fir nonstructural carbohydrates using a different extraction procedure. Therefore, we do not report nonstructural carbohydrate data for subalpine fir.

### Glucose and Sucrose

We added 20 μl of the ethanol extract of each sample to three wells for technical replicates. We evaporated the ethanol in a drying oven at 55°C for 20 minutes. We then dissolved the extract in 20 μl H_2_0. Samples being analyzed for sucrose were treated with 10 μl of invertase and incubated at 37°C for 10 minutes. We then added 200 μl GOPOD reaction mix (Glucose oxidase/peroxidase reagent with O-Dianisidine) (Total Starch Assay Kit, Megazyme Co., Wicklow, Ireland) to the standards, and glucose, and sucrose sample wells. We incubated the samples for 20 minutes at ambient temperature and read absorbance at 510 nm using a spectrophotometer. Concentrations were determined from glucose and sucrose standard curves. The results for glucose and sucrose were summed and presented as percentage of dry matter.

### Starch

In order to quantify foliar concentrations of starch, we modified the enzymic procedure from Hendrix 1993 [31] by adapting it to the total starch assay procedure from the Megazyme starch extraction kit. Ethanol extracted leaf tissue was digested in 1 ml of α-amylase. Samples were then boiled for 20 minutes and subsequently cooled for 10 minutes. 15 μl amyloglucosidase was then added to each sample and samples were placed in a shaking water bath at 50°C for 45 minutes. Samples were then plated out and 200 μl of GOPOD reaction mix was added to the samples. After 20 minutes at room temperature, absorbance was read at 510 nm on the spectrophotometer. We quantified sample starch concentrations using a starch standard curve from the Megazyme maize starch control at 20, 10, 5, 2.5 and 0 μg / 20 μl concentrations. We ran a positive control sample from aspen leaf tissue with known concentrations of glucose, sucrose and starch to verify that the protocol was working correctly. The results were presented as percentage of dry matter.

### Statistical Analysis

Water relations, foliar nutrients, defense chemicals, and nonstructural carbohydrates were tested for significant differences across stand type, proximity, and size class using linear regression models with random slopes across locations. We used a top-down strategy of model selection with REML (restricted maximum likelihood) estimation [32] following a model selection protocol by Zuur et al 2009 [33]. This method first finds an optimal random effects model, using a global model, before evaluating fixed effects. We then used F-statistics to test fixed effects. In order to confirm assumptions of homogeneity of variance we compared fitted values to standardized residuals after finding random slope structures and then again, once optimal fixed effects were identified.

If a model contained one or more significant variables, we then conducted F-protected pairwise Tukey adjusted t-tests (at *p* = 0.05) to test across stand composition, proximity and size class for both aspen and subalpine fir. Statistical significance was reported at *p* ≤ 0.05 for these multiple comparisons. F-values are presented in the tables and results section, while p-values of pairwise tests are denoted by letters on the tables. We tested each variable for normality using visual inspection of a Q-Q plot and a histogram of standardized residuals. We square root transformed condensed tannins and phosphorus for analysis and then back-transformed them for presentation. Aspen and subalpine fir were analyzed separately. Only aspen was evaluated for nonstructural carbohydrates and phenolic glycosides. All analyses were conducted using the program R [34], and packages nlme, MuMIn, multcomp [35, 36, 37]

## Results

### Water Relations

Stand type, proximity class, and size class had no significant effects on stem water potential for either species (Tables 1 and 2).

**Table 1:** Aspen foliar leaf physiology as a function of stand type, proximity & size class.

Treatments	Starch %dw	Glucose+Sucrose % dw	Water pot. (MPa)	N %dw	P %dw	Phenolic Glyc. % dw	Tannins % w
**Stand Type**							
Aspen Stand	1.1 ± 0.12ab	2.6 ± 0.17	-1.8 ± 0.09	2.0 ± 0.04	0.35 ± 0.02	9.3 ± 0.83	3.6 ± 0.38
Mixed Stand	1.0 ± 0.12b	2.3 ± 0.18	-1.5 ± 0.11	2.0 ± 0.05	0.37 ± 0.02	7.9 ± 0.63	4.4 ± 0.44
Conifer Stand	1.2 ± 0.09a	2.3 ± 0.19	-1.9 ± 0.10	2.1 ± 0.05	0.38 ± 0.03	8.8 ± 0.92	3.5 ± 0.35
**Proximity**							
Proximate	1.0 ± 0.08a	2.3 ± 0.16	-1.7 ± 0.09	2.0 ± 0.02	0.37 ± 0.03	8.4 ± 0.65	4.3 ± 0.39
Independent	1.2 ± 0.08b	2.6 ± 0.13	-1.7 ± 0.08	2.0 ± 0.02	0.35 ± 0.02	8.9 ± 0.64	3.5 ± 0.27
**Size class**							
Small (15–19cm)	0.9 ± 0.12	2.1 ± 0.19a	-1.7 ± 0.11	2.0 ± 0.05	0.36 ± 0.03	7.9 ± 0.80	4.5 ± 0.50
Medium (20–24cm)	1.1 ± 0.14	2.5 ± 0.17b	-1.6 ± 0.10	2.0 ± 0.04	0.39 ± 0.02	9.9 ± 0.77	3.7 ± 0.35
Large (25–30cm)	1.3 ± 0.17	2.8 ± 0.16c	-1.8 ± 0.10	2.0 ± 0.06	0.33 ± 0.02	7.9 ± 0.75	3.5 ± 0.39
F-value, *p*-value							
**Stand Type**	F = 10, *p* < 0.001	ns	ns	ns	ns	ns	ns
**Paired**	F = 9.8, *p* = 0.002	ns	ns	ns	ns	ns	ns
**Size class**	ns	F = 6.8, *p* = 0.002	ns	ns	ns	ns	ns

Aspen functional traits as a function of stand type, proximity & size class. Aspen stands were 90% aspen overstory, mixed stands were 50% aspen and 50% conifer, and conifer stands were 90% conifer. Proximate aspen trees were < 20 cm from a subalpine fir and independent aspen trees were growing > 3 m from a subalpine fir or other aspen trees. Size classes were measured in diameter at breast height (1.4 m). Means presented ± standard error. ns = nonsignificant result. Statistical differences among pairwise comparisons at *p* = 0.05 are denoted by letters. All units are in percent dry weight (% dw), except for stem water potential, which is presented in megapascals.

**Table 2:** Subalpine foliar leaf physiology as a function of stand type, proximity, & size class.

Treatments	Water Pot. (MPa)	P % dw	N % dw	Tannins % dw
**Stand Type**				
Aspen Stand	-1.1 ± 0.07	0.27 ± 0.01a	0.87 ± 0.03	4.2 ± 0.18
Mixed Stand	-1.2 ± 0.09	0.25 ± 0.01a	0.90 ± 0.03	4.8 ± 0.22
Conifer Stand	-1.2 ± 0.09	0.29 ± 0.01a	0.90 ± 0.04	4.4 ± 0.24
**Proximity**				
Proximate	-1.2 ± 0.07	0.27 ± 0.01	0.89 ± 0.04	4.6 ± 0.21
Independent	-1.2 ± 0.07	0.27 ± 0.01	0.89 ± 0.03	4.3 ± 0.19
**Size class**				
Small (15–19cm)	-1.2 ± 0.08	0.27 ± 0.01	0.94 ± 0.4	4.8 ± 0.23
Medium (20–24cm)	-1.2 ± 0.08	0.28 ± 0.01	0.83 ± 0.4	4.2 ± 0.18
Large (25–30cm)	-1.1 ± 0.10	0.26 ± 0.01	0.91 ± 0.3	4.4 ± 0.22
F-value, *p*-value				
**Stand Type**	ns	F = 5.4, *p*<0.001	ns	ns
**Paired**	ns	ns	ns	ns
**Size class**	ns	ns	ns	ns

Subalpine fir functional traits as a function of stand type, proximity & size class. Aspen stands were 90% aspen overstory, mixed stands were 50% aspen and 50% conifer, and conifer stands were 90% conifer. Proximate fir trees were < 20 cm from an aspen tree and independent fir trees were growing > 3 m from aspen or fir trees. Size classes are measured in diameter at breast height (1.4 m). Means presented ± standard error. ns = nonsignificant result. All units are in percent dry weight (% dw), except for stem water potential, which is presented in megapascals.

### Foliar nutrients–P, N

Foliar concentrations of phosphorus in aspen were not significantly different based on stand type, proximity and size class. There was no significant effect of stand type, proximity and size class on foliar N concentrations in aspen (Table 1).

Foliar phosphorus concentrations in subalpine fir leaves did not vary significantly across stand type, size classes or by proximity (Table 2). Foliar nitrogen concentrations in subalpine fir were not significantly influenced by stand type, size class, or by proximity (Table 2).

### Defense Chemistry–Condensed Tannins, Phenolic Glycosides

Phenolic glycosides and condensed tannin levels in aspen leaves did not vary significantly by stand type, proximity, or size classes (Table 1). Foliar concentrations of condensed tannins of subalpine fir trees did not vary by stand type, size class, or by proximity (Table 2).

### Carbohydrates–Glucose, Sucrose and Starch

Aspen foliar starch levels were 17% lower when growing in close proximity to subalpine fir compared to independently growing aspen trees (F = 9.8, *p* = 0.002) (Table 1). Aspen foliar starch levels were 20% higher in conifer dominated stands compared to mixed stands and 9% higher than aspen dominated stands (F = 10, *p* = 0.001) (Table 1). Tree size was not significantly related to foliar starch levels of aspen leaves.

In aspen, foliar concentrations of glucose + sucrose were 12% and 33% higher in the largest size class compared to the intermediate and small size classes, respectively (F = 6.8, *p* = 0.002) (Table 1). Stand type and proximity had no significant impact on glucose + sucrose concentrations (Table 1).

## Discussion

The effects of stand composition, proximity and tree size on leaf traits tended to be nonsignificant or weak, which was surprising considering the scope of community composition and structure gradients. The general lack of leaf functional response suggests functional coexistence between aspen and subalpine fir across these gradients. In our system, aspen may share nutrients, water, and carbohydrates through clonal integration of roots [38, 39, 40] which may buffer against competition driven resource limitation [41] due to increasing dominance of subalpine fir and its close proximity to aspen [18]. Functional sensitivity to interspecific competition by aspen may also be muted by the resource storage ability of aspen [42].

Conifer and deciduous species have been shown to intermix without affecting the productivity of each other [43], and in mixed boreal forests white spruce (*Picea glauca* (Moench)) productivity was increased in aspen stands, and intermixing did not affect aspen growth [44]. While our study was conducted on a limited spatial and temporal scale, our results suggest that leaf function in mature aspen and subalpine fir do not vary strongly across gradients of stand composition, proximity or tree size.

### Stand Composition

Disturbance can increase biodiversity, but some species may be excluded if disturbance becomes too infrequent [45]. Aspen are thought to depend on fire to reduce conifer dominance [21, 46], which promotes unfavorable light and soil conditions for aspen. Aspen saplings are sensitive to shade and changes in soil chemistry that develop in later stages of forest succession, characterized by conifer dominance [8]. We expected that previously observed aspen sapling sensitivity to conifer dominance would extend to mature aspen trees (16), but our data suggest that mature aspen are not as sensitive as saplings to increasing conifer abundance. This resilience to changing stand composition may be a function of aspen’s resource storage in its root system [42, 47], and resource sharing due to clonal integration [47, 41]. Resource storage can stabilize niche differences [48], which promotes coexistence in the presences of competitors when resources vary in space and time [49], such as the highly variable precipitation patterns that occurred before our study.

While many studies have found strong differences in leaf function and physiology of aspen and subalpine fir juveniles along gradients of biotic and abiotic stress (stand composition, light and soil environments, or topography) [8, 50, 51], these findings may not extend to leaf function in adult trees. In broadleaf species, overstory trees with leaves exposed to the canopy have shown similar leaf function despite interspecific differences in shade tolerances [52]. This suggests that the upper canopy foliage of adult trees are less affected by neighboring trees than understory trees. Also, the conical growth form of subalpine fir may produce less interspecific and intraspecific effects on neighboring species of similar height [53]. In general, conical crowns allow light to penetrate into stands [54] which may limit shade effects for nearby adult trees. While foliar starch levels in aspen were significantly higher in conifer versus aspen stands the difference was slight and may not be biologically significant. Access to sufficient light in the canopy by adult trees, and the compact growth form of subalpine fir could explain the weak response to gradients of stand composition for adult trees in our study.

### Proximity

While competition varies along gradients of soil fertility [55], light [56–59], and water availability [60], the strength of these competitive effects are also influenced by distance relationships between plants [1, 12, 61]. Previous work shows that proximity of mature aspen to subalpine fir correlates to higher mortality of aspen [16] and aspen sapling function is reduced when grown in soils and light environments that have been modified by high conifer abundance [7, 8].

Our hypothesis that proximity reduces stem water potential and foliar concentrations of defense and nonstructural carbohydrates was generally unsupported by our data. If trees are not resource limited due to adequate moisture, for example, then competitive interactions may be minimized [61]. The high precipitation of July 2012 (nearly 300% over the 30 year average), and lack of differences in stem water potential between proximate and independently growing trees suggest that sufficient water resources may have limited competition for soil water. Further, aspen’s clonal nature could buffer individual stems from general resource limitation (light, nutrients, water) and competition [41, 47]. However, we did find that mature aspen growing next to subalpine fir had 17% lower levels of foliar starch (Table 1), an important biomarker for tree vigor [24]. Lower foliar starch storage suggests that close proximity to subalpine fir may create some antagonism for aspen carbon metabolism, but the effect size was small which can be problematic in terms of ecological interpretation [62]. These results suggest that stabilizing niche differences possibly caused by resource storage in aspen, could contribute to aspen-subalpine fir coexistence [42, 48]. However, while we show short term coexistence, this relationship likely changes at larger time scales. Long-term processes that exclude aspen during succession to subalpine fir could be punctuated events of stress, such as multi-year drought, or long-term depression of resource uptake driven by competition.

Using the carbon-nutrient balance theory as a framework, we anticipated that subalpine fir would be tolerant of competition with aspen. Close proximity to aspen had no significant effect on the leaf functional traits we measured for subalpine fir. Subalpine fir is a strong competitor among conifer species [63] and competitive effects on subalpine fir may be more influenced by intraspecific vs interspecific competition [63]. Further, the density of competing neighbors may be more important than one competitor at a short distance [53]. Therefore, subalpine fir’s insensitivity to a proximate aspen may be a due to the low plasticity of functional traits common to climax species, subalpine fir’s strong competitive ability, or perhaps light conditions did not differ considerably across the gradients of proximity we measured.

### Size Class

As plants increase in size, they generally increase their resource use and their zone of influence in plant-plant interactions [16]. We asked whether leaf functional traits vary depending on tree size. As the size of paired trees increased we expected stronger competitive effects. We found little evidence that leaf resource acquisition and function varied across gradients of tree size and there was no evidence of interactions between size, proximity relationships or stand composition. The general lack of support for our hypothesis may also be due to low phenotypic plasticity of leaf traits at later stages of aspen tree development [64]. Beyond the juvenile stage, leaf functional traits tend to be similar across different size classes in aspen [64]. Similarly, mature subalpine fir trees have shown resilience to variable resource environments along gradients of temperature and elevation [65].

While some studies indicate that neighboring tree size and distance are important in determining the magnitude of effect of competition on adult trees [66], other studies suggest that the density of neighboring trees has a stronger impact [53, 63]. We show that size class of aspen or subalpine fir had no main effects or interactive effects across stand type or proximity.

## Conclusion

In conclusion, we found few differences in physiology and function of aspen and subalpine fir leaf traits, which is surprising given the gradients of stand composition, proximity and size we explored here. These results suggest that mechanisms of coexistence allow both aspen and subalpine fir to maintain leaf function across a wide range of stand structural characteristics. The intermixing of aspen and subalpine fir across gradients of stand composition, proximity and tree sizes provides evidence of functional coexistence of these long-lived perennials.
